# Nrf2 deficiency promotes the increasing trend of autophagy during aging in skeletal muscle: a potential mechanism for the development of sarcopenia

**DOI:** 10.18632/aging.102990

**Published:** 2020-04-03

**Authors:** Dong-Dong Huang, Xia-Lin Yan, Sheng-Dong Fan, Xi-Yi Chen, Jing-Yi Yan, Qian-Tong Dong, Wei-Zhe Chen, Na-Xin Liu, Xiao-Lei Chen, Zhen Yu

**Affiliations:** 1 Department of Gastrointestinal Surgery, The First Affiliated Hospital of Wenzhou Medical University, Wenzhou, China; 2 Department of Gastrointestinal Surgery, Shanghai Tenth People’s Hospital Affiliated to Tongji University, Shanghai, China; 3 The Second People’s Hospital of Wuhu, Wuhu, China; 4 Department of Cardiothoracic Surgery, The Second Affiliated Hospital of Wenzhou Medical University, Wenzhou, China; 5 Department of Pancreatitis Center, The First Affiliated Hospital of Wenzhou Medical University, Wenzhou, China

**Keywords:** sarcopenia, autophagy, aging, skeletal muscle, autophagy flux measurements

## Abstract

This study aims to explore the impact of nuclear factor erythroid 2-related factor 2 (Nrf2) deficiency on skeletal muscle autophagy and the development of sarcopenia. LC3b, P62, Bnip3, Lamp-1, and AMPK protein levels were measured in muscle from young, middle-aged, old Nrf2−/− (knockout, KO) mice and age-matched wild-type (WT) C57/BL6 mice. Autophagy flux was measured in young WT, young KO, old WT, old KO mice, using colchicine as autophagy inhibitor. There was a trend of higher accumulation of LC3b-II, P62, Bnip3, Lamp-1 induced by colchicine in old WT mice compared with young WT mice. Colchicine induced a significantly higher accumulation of LC3b-II, P62, Bnip3, Lamp-1 in KO mice compared with WT mice, both in the young and old groups. AMPK and reactive oxygen species (ROS) were unregulated following Nrf2 KO and increasing age, which was consistent with the increasing trend of autophagy flux following Nrf2 KO and increasing age. Nrf2 KO and increasing age caused decreased cross-sectional area of extensor digitorum longus and soleus muscles. We concluded that Nrf2 deficiency and increasing age may activate AMPK and ROS signals to cause excessive autophagy activation in skeletal muscle, which can be a potential mechanism for the development of sarcopenia.

## INTRODUCTION

Autophagy is an evolutionary conserved housekeeping cellular degradation and recycling process, whereby misfolded proteins and exhausted organelles are degraded to maintain cellular homeostasis. A series of autophagy-related proteins are involved in these processes, including microtubule associated protein 1 light chain 3-II (LC3-II), LC3-I, sequestosome 1 (P62), BCL2/adenovirus E1B interacting protein 3 (Bnip3), and lysosomal-associated membrane protein 1 (Lamp-1) [1, 2].

Skeletal muscle is the most abundant tissue in human body, accounting for about 40-55% of the body weight. It is also the largest metabolic organ in the body [1]. Autophagy plays a key role in the regulation of muscle mass, either excessive or impaired autophagy leads to muscle mass wasting [3]. Deficiency in the basic autophagy function causes accumulation of misfolded proteins and exhausted organelles and results in skeletal muscle cell dysfunction and death. On the contrary, excessive autophagy can also be deleterious by causing cellular stress and muscle protein degradation [4].

Muscle mass declines with increasing age, which is termed sarcopenia [5]. At the cellular level, aging is characterized by a progressive accumulation of dysfunctional cellular proteins and organelles, which results in a disruption of cellular homeostasis, progressive degeneration, and increases the risk of cell death [6, 7]. Several animal models have showed that ablation of autophagy function results in precocious aging and muscle wasting in mice [8, 9]. However, it is still controversial about the changes of autophagy function in skeletal muscle with increasing age. Based on the evidence from lower organisms and non-muscle tissue, most literature held the concept that skeletal muslcle autophagy declines with aging. Declined autophagy results in the accumulation of dysfunctional and damaged cellular components and causes muscle cells impairments and muscle wasting [10, 11]. On the contrary, a recent study showed that autophagy and mitophagy in mice muscle were enhanced during aging, which may contribute to the decline in organelle contents and muscle mass, but serve to maintain a healthy organelle pool and muscle cells function [12]. Many studies have shown that the expression of autophagy-related proteins in muscle alters significantly during aging, but inconsistent results have been found from different studies. McMullen et al. showed that the expression of Atg5, Atg7, LC3-II, and LC3-I decreased during aging in rat skeletal muscle [13]. On the contrary, Komatsu et al. found that the expression of LC3 increased during aging in mice muscle, whereas the ratio of LC3-II/LC3-I remained unaltered [14]. Moreover, a study from Sebastian et al. showed that Bnip3, P62, LC3-II, and LC3-II/LC3-I increased during aging in mice muscle [11]. These inconsistent results suggested that the measurement of autophagy-related proteins at the static level can often lead to discrepancies in interpretation, because autophagy is a dynamic process. Therefore, measurement of autophagy flux is necessary to reflect the real condition of autophagy within muscle cells during aging. Until a recent study, no previous studies have investigated the alterations of autophagy during aging in skeletal muscle using autophagy flux measurements [12].

Nuclear factor erythroid 2-related factor 2 (Nrf2) is a cytoprotective gene which mainly functions to protect cells against oxidative stress and toxicants. In recent years, increasing evidences have revealed the role of Nrf2 in the regulation of autophagy [15, 16]. However, few studies have investigated the role of Nrf2 in regulation of autophagy in skeletal muscle. Previous studies from our group and other researchers have found that deficiency of Nrf2 exacerbated muscle loss in the old individuals [17, 18]. However, it is still unclear about the alterations of autophagy after genetic ablation of Nrf2. Therefore, the present study aims to explore the impact of Nrf2 deficiency on the autophagy function in skeletal muscle, as well as its connection with the development of sarcopenia during aging.

## RESULTS

### Influence of Nrf2 deficiency and increasing age on the expression of autophagy-related proteins in skeletal muscle

**Figure 1:**
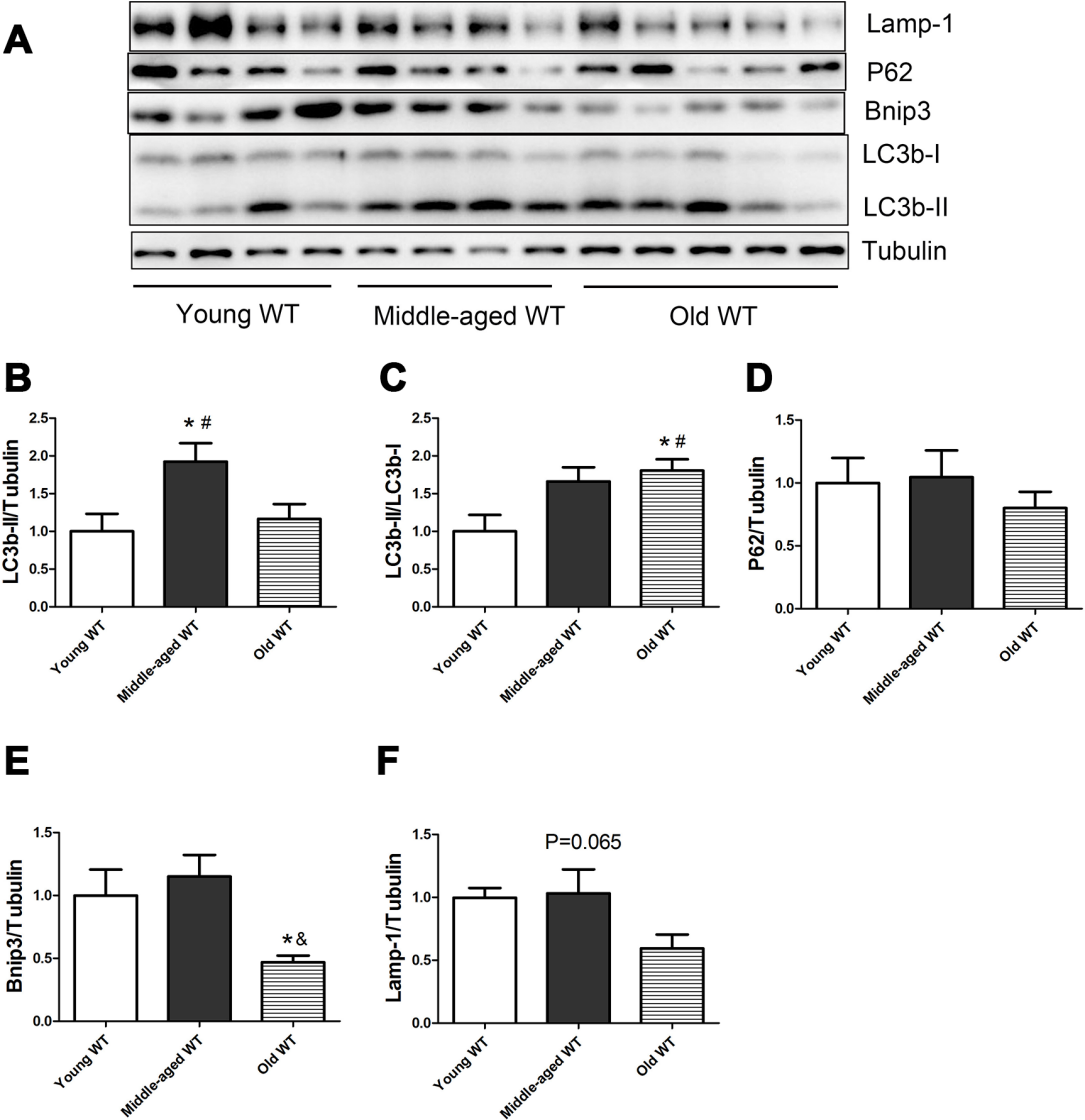
**Expression of LC3b-II, LC3b-I, P62, Bnip3, and Lamp-1 proteins in young WT, middle-aged WT, and old WT mice.** (**A**) Western blot images. (**B**–**F**) Statistical graphs. Data represent mean ± SE, (n=4-5). *P <0.05 effect of age by one-way ANOVA. #P <0.05 compared with young WT mice. &P <0.05 compared with middle-aged WT mice.

As shown in Figure 1, in WT mice, the expression of LC3b-II and the ratio of LC3b-II/LC3b-I increased during aging (P <0.05). Middle-aged WT mice had a significantly higher LC3b-II level compared with young WT mice (P <0.05). Old WT mice had a significantly higher LC3b-II/LC3b-I ratio compared with young WT mice (P <0.05). P62 did not show significant alterations during aging. Bnip3 protein level decreased significantly during aging. Old WT mice had a significantly lower Bnip3 level compared with middle-aged WT mice. Lamp-1 showed a trend of decrease during aging, but the difference was not statistically significant.

**Figure 2:**
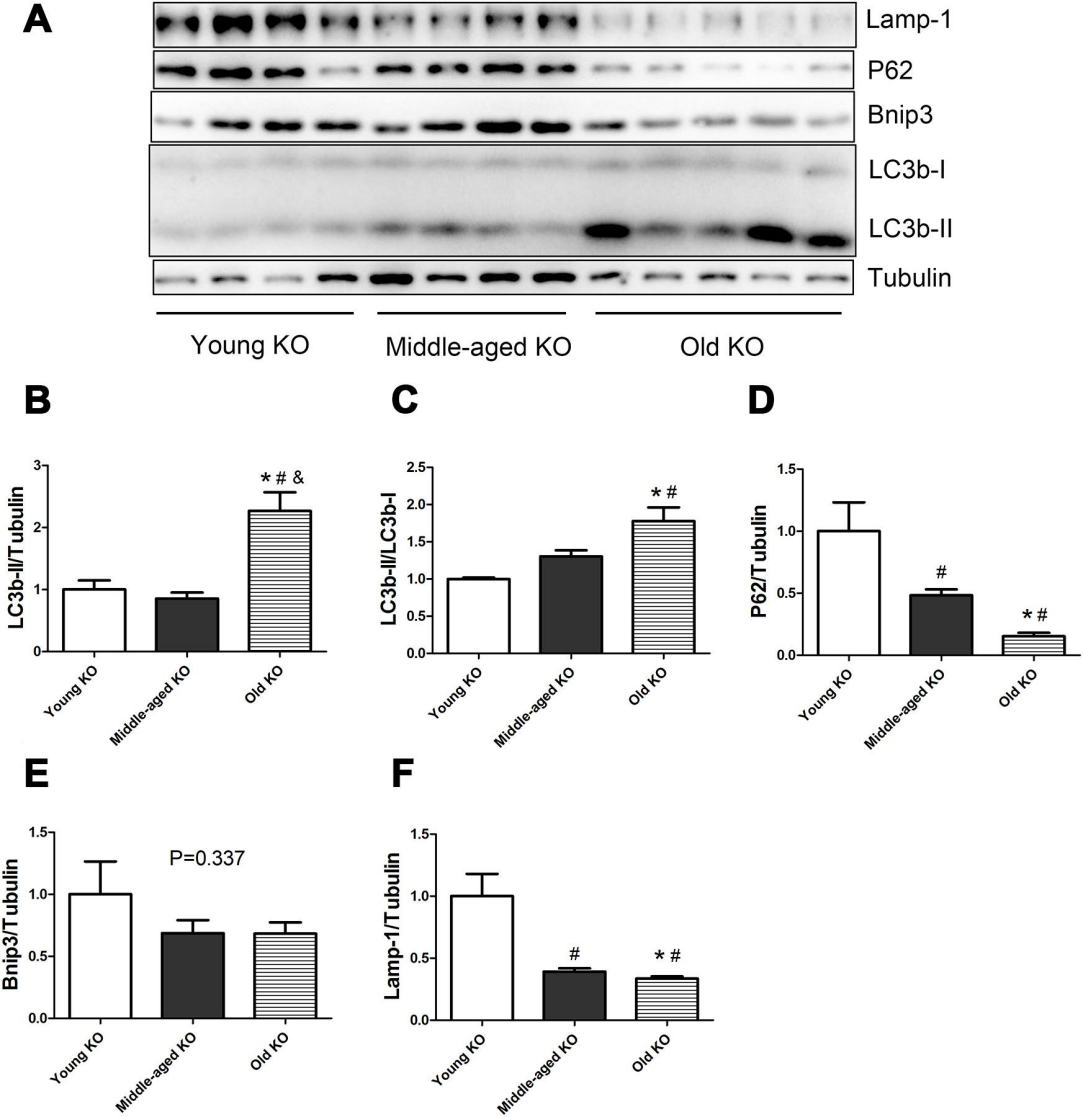
**Expression of LC3b-II, LC3b-I, P62, Bnip3, and Lamp-1 proteins in young KO, middle-aged KO, and old KO mice.** (**A**) Western blot images. (**B**–**F**) Statistical graphs. Data represent mean ± SE, (n=4-5). *P <0.05 effect of age by one-way ANOVA. #P <0.05 compared with young KO mice. &P <0.05 compared with middle-aged KO mice.

As shown in Figure 2, in Nrf2 KO mice, the expression of LC3b-II and the ratio of LC3b-II/LC3b-I increased during aging (P <0.05). Old KO mice had a significantly higher expression of LC3b-II compared with young KO and middle-aged KO mice. Moreover, old KO mice had a significantly higher LC3b-II/LC3b-I ratio compared with young KO mice. P62 protein level decreased significantly with aging in KO mice. Middle-aged KO and old KO mice had a significantly lower P62 level compared with young KO mice. Bnip3 protein level showed a trend of decrease with aging in KO mice, but the difference was not statistically significant. Lamp-1 protein level decreased significantly with aging in KO mice. Middle-aged KO and old KO mice had a significantly lower Lamp-1 level compared with young KO mice.

**Figure 3:**
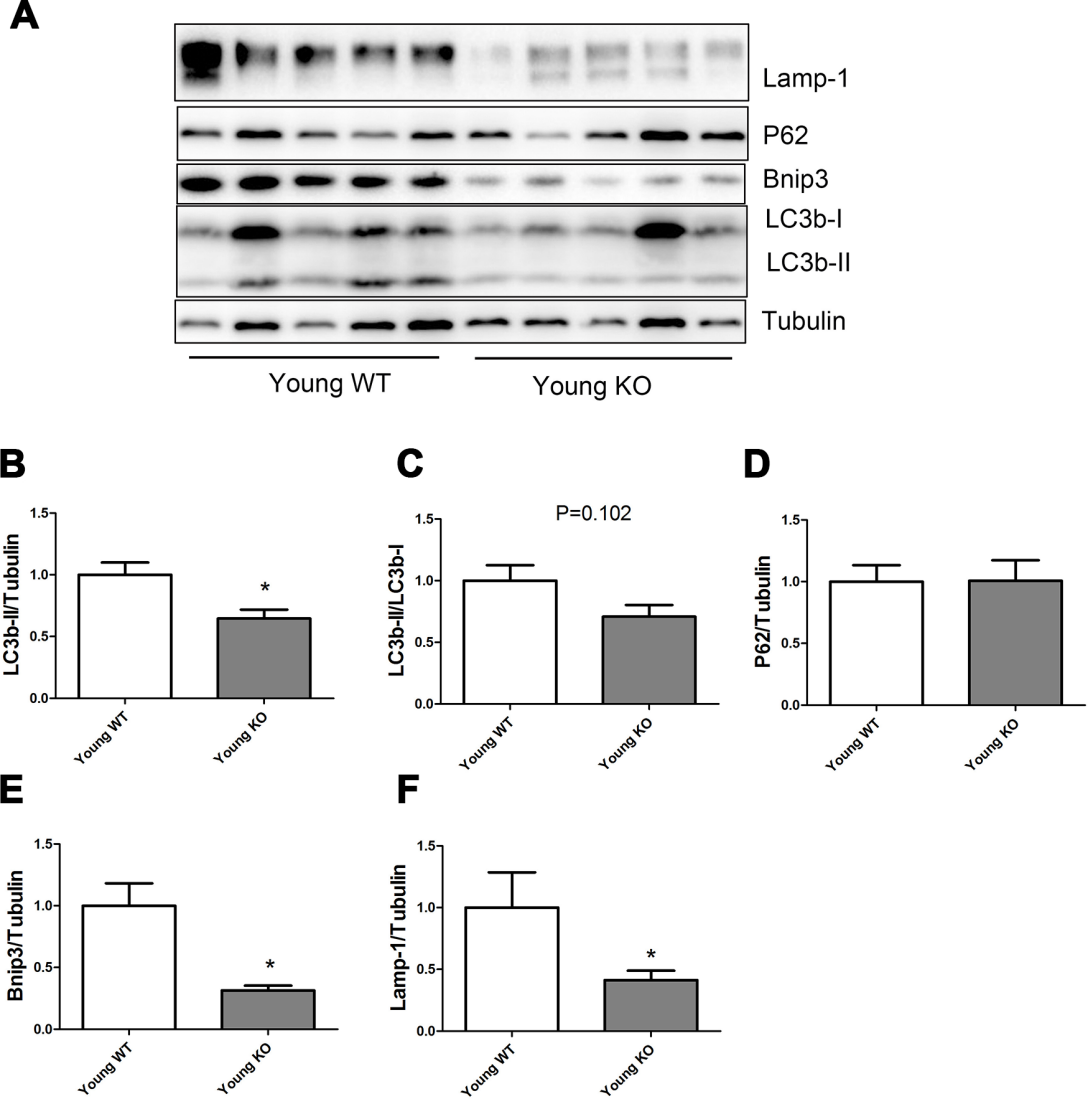
**Expression of LC3b-II, LC3b-I, P62, Bnip3, and Lamp-1 proteins in young WT and young KO mice.** (**A**) Western blot images. (**B**–**F**) Statistical graphs. Data represent mean ± SE, (n=5), *statistically significant.

As shown in Figure 3, Nrf2 deficiency caused a significant decrease of LC3b-II, Bnip3, and Lamp-1 levels in the young mice, but did not seem to influence the P62 expression level. LC3b-II/LC3b-I ratio showed a trend of decrease in young KO mice compared with young WT mice, but the difference was not statistically significant.

**Figure 4:**
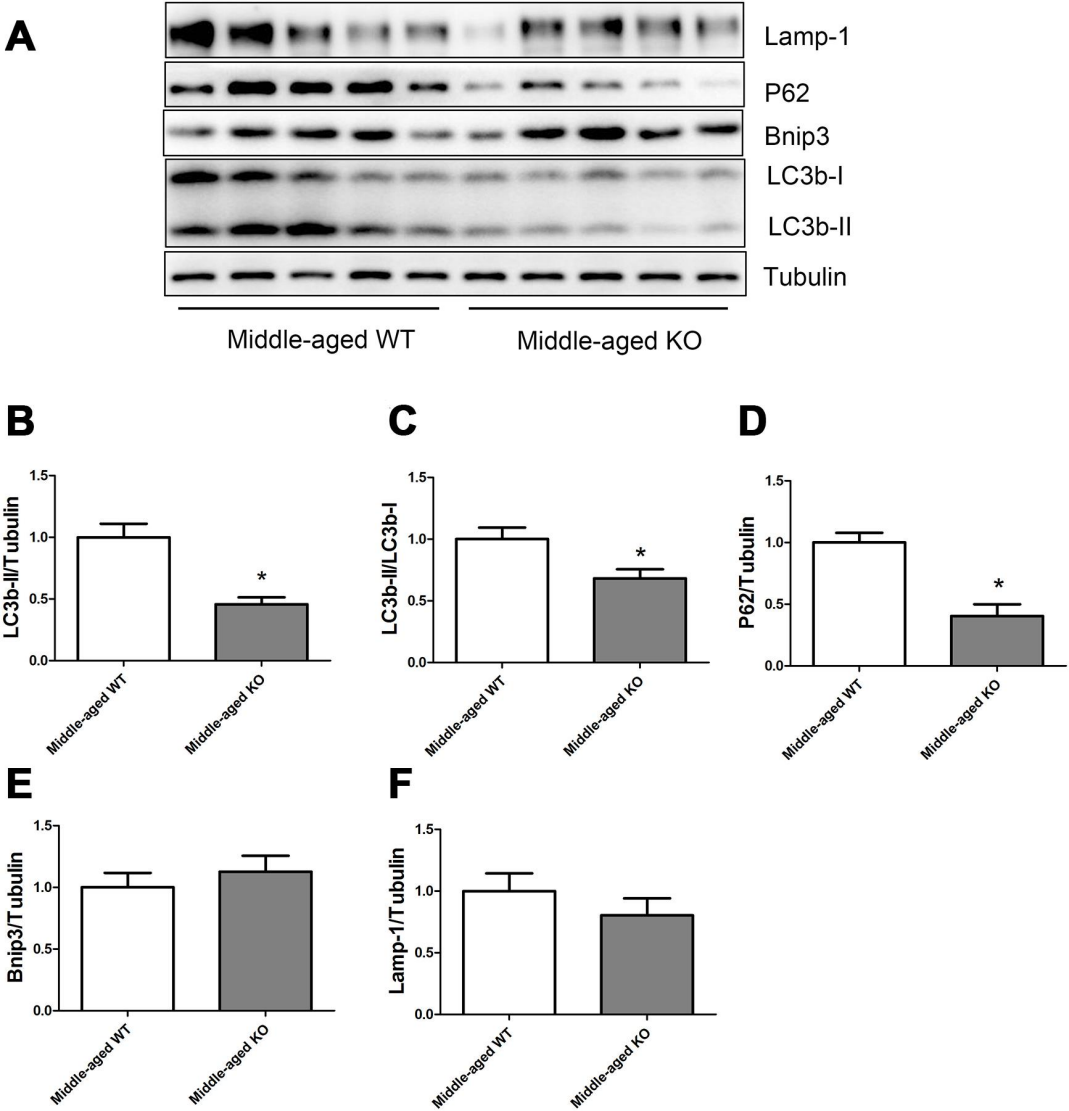
**Expression of LC3b-II, LC3b-I, P62, Bnip3, and Lamp-1 proteins in middle-aged WT and middle-aged KO mice.** (**A**) Western blot images. (**B**–**F**) Statistical graphs. Data represent mean ± SE, (n=5), *statistically significant.

As shown in Figure 4, Nrf2 deficiency caused a significant decrease of LC3b-II, LC3b-II/LC3b-I ratio, and P62 levels, but did not seem to influence the Bnip3 and Lamp-1 levels in the middle-aged mice.

**Figure 5:**
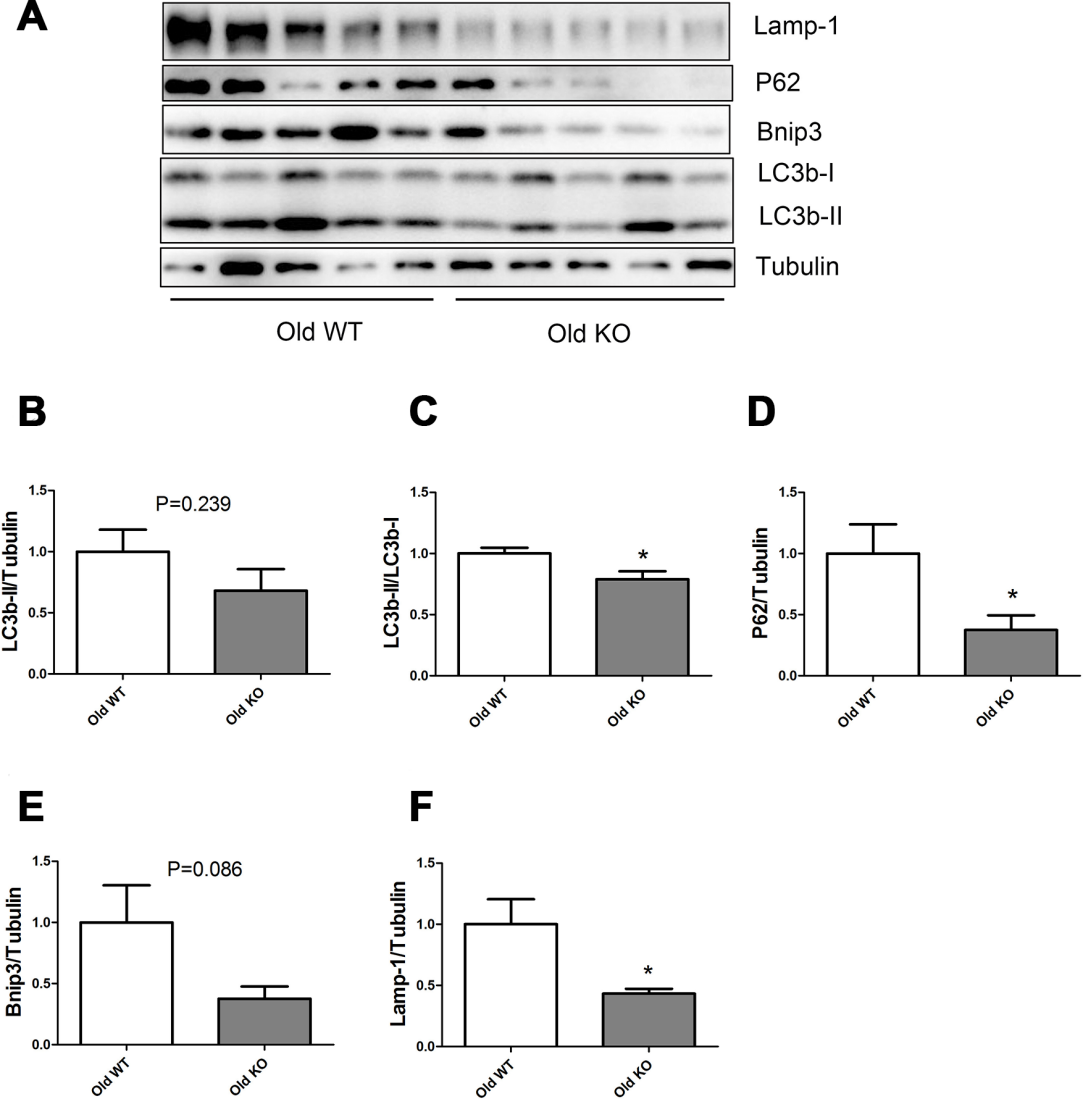
**Expression of LC3b-II, LC3b-I, P62, Bnip3, and Lamp-1 proteins in old WT and old KO mice.** (**A**) Western blot images. (**B**–**F**) Statistical graphs. Data represent mean ± SE, (n=5), *statistically significant.

As shown in Figure 5, Nrf2 deficiency caused a trend of decrease in LC3b-II and Bnip3 in the old mice, but the difference was not statistically significant. Old KO mice had a significantly lower LC3b-II/LC3b-I ratio, P62, and Lamp-1 levels compared with old WT mice.

### Nrf2 deficiency promoted the increase of autophagy flux induced by increasing age

**Figure 6:**
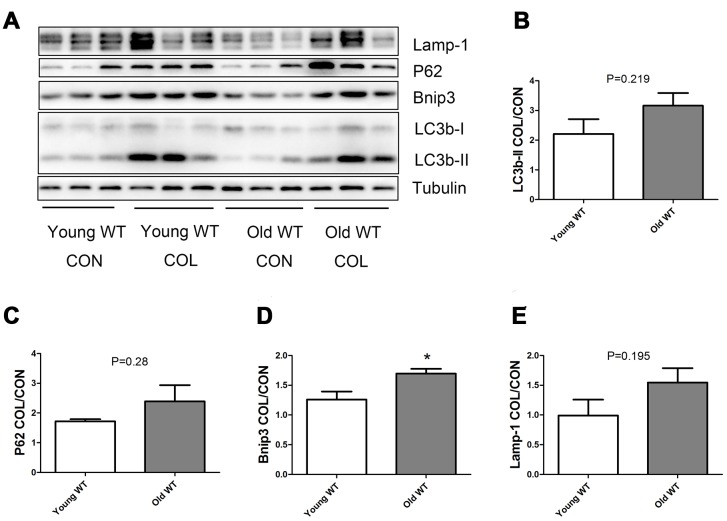
**Autophagy flux in skeletal muscle of young WT and old WT mice.** (A) Western blot images. (**B**–**E**) Statistical graphs. Autophagy flux was calculated by the fold of changes in the expression of LC3b-II, P62, Bnip3, and Lamp-1 induced by colchicine. Data represent mean ± SE, n=3. *statistically significant.

**Figure 7:**
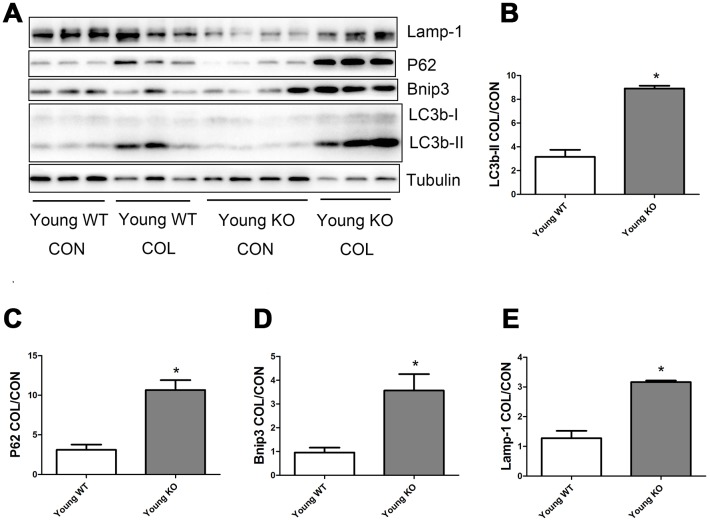
**Autophagy flux in skeletal muscle of young WT and young KO mice.** (**A**) Western blot images. (**B**–**E**) Statistical graphs. Data represent mean ± SE, n=3-4. *statistically significant.

**Figure 8:**
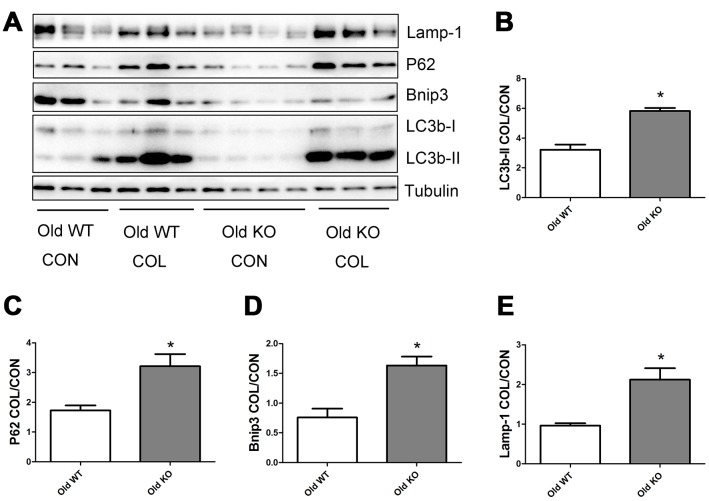
**Autophagy flux in skeletal muscle of old WT and old KO mice.** (**A**) Western blot images. (**B**–**E**) Statistical graphs. Data represent mean ± SE, n=3-4. *statistically significant.

As shown in Figure 6, old WT mice had a trend of higher colchicine-induced accumulation of LC3b-II, P62, and Lamp-1 compared with young WT mice, but the difference were not statistically significant. Colchicine induced a significantly higher accumulation of Bnip3 in the old WT mice compared with young WT mice (P <0.05). As shown in Figure 7, colchicine induced a significantly higher accumulation of LC3b-II, P62, Bnip3, and Lamp-1 in the young KO mice compared with young WT mice (P <0.05). Similarly, colchicine induced a significantly higher accumulation of LC3b-II, P62, Bnip3, and Lamp-1 in the old KO mice compared with old WT mice (P <0.05) (Figure 8).

### Nrf2 deficiency and increasing age activated AMPK and ROS signals

**Figure 9:**
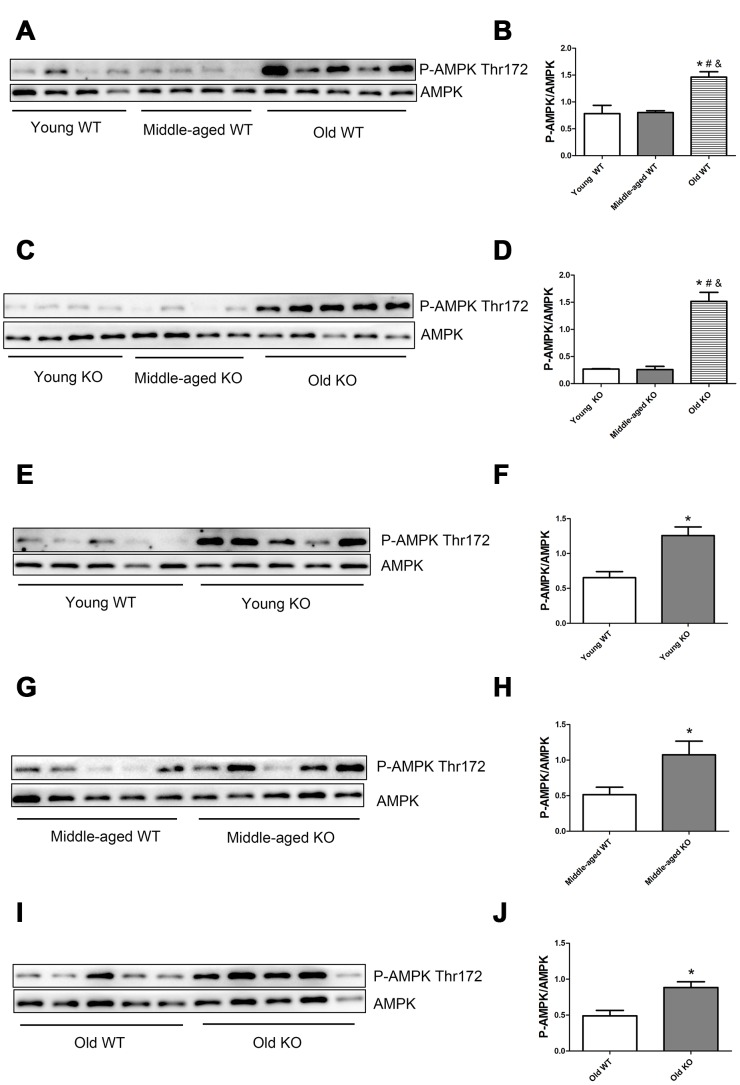
**AMPK phosphorylation level in skeletal muscle of different groups.** (**A**, **C**, **E**, **G**, **I**) Western blot images. (**B**, **D**, **F**, **H**, **J**) Statistical graphs. Data represent mean ± SE, n=4-5. *P <0.05 main effect. #P <0.05 compared with young mice. &P <0.05 compared with middle-aged mice.

**Figure 10:**
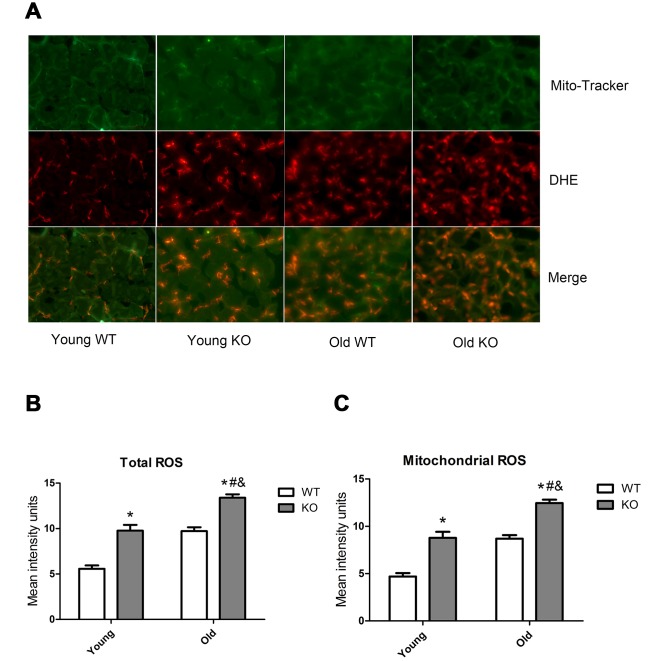
**ROS level in skeletal muscle of different groups.** (**A**) Mito-Tracker green is a marker of mitochondria, dihydroethidium (DHE) is a probe of reactive oxygen species (ROS). Images from green and red fluorescence were merged (yellow) to locate the mitochondrial source of ROS generation. The yellow color indicates ROS within the mitochondria. (**B**) Total ROS. (**C**) Mitochondrial ROS. Data represent mean ± SE, n=3. #P <0.05 main effect of age by two-way ANOVA. &P <0.05 main effect of genotype by two-way ANOVA.*P <0.05 Nrf2 KO vs WT mice of the same age.

AMP-activated protein kinase (AMPK) and reactive oxygen species (ROS) are well-recognized activator of autophagy. As shown in Figure 9, AMPK phosphorylation level in the threonine (Thr) 172 site increased significantly during aging, both in the WT and KO mice. Moreover, Nrf2 deficiency caused a significant increase of AMPK Thr172 phosphorylation level in all of the three age groups. As shown in Figure 10, total ROS and mitochondrial ROS levels increased significantly during aging, both in the WT and KO mice. Moreover, Nrf2 deficiency caused a significant increase of total ROS and mitochondrial ROS levels both in the young and old mice.

### Nrf2 deficiency exacerbated skeletal muscle loss during aging

**Figure 11:**
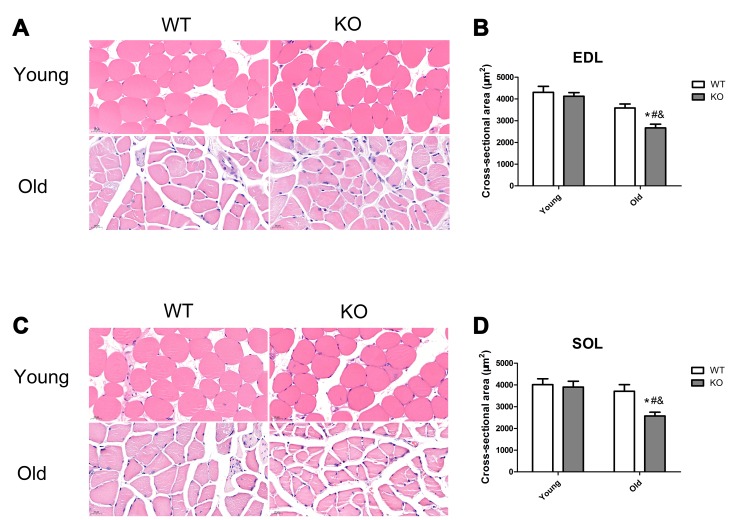
**Nrf2 deficiency exacerbated skeletal muscle loss during aging.** (**A**, **C**) Representative images of the cross-sectional area (CSA) of extensor digitorum longus (EDL) and soleus (SOL) muscles by HE staining. (**B**, **D**) CSA of the EDL and SOL muscles in the young and old mice of WT and KO genotypes. Data represent mean ± SE, n=3. #P <0.05 main effect of age by two-way ANOVA. &P <0.05 main effect of genotype by two-way ANOVA.*P <0.05 Nrf2 KO vs WT mice of the same age.

Skeletal muscle mass was analyzed by measurement of CSA in the EDL and SOL muscles. As shown in Figure 11, Nrf2 deficiency and increasing age caused a significant decrease of CSA both in the EDL and SOL muscles (P <0.05). Moreover, Nrf2 deficiency resulted in a significant decreased CSA of the EDL and SOL muscles in the old mice (P <0.05), but not in the young mice.

## DISCUSSION

Currently, most literature held the opinion that autophagy function decreased during aging [10, 11]. However, most of these evidences were based on measurements of autophagy-related genes/proteins at. the static level, which can often lead to discrepancies in interpretation. It is has been recognized that autophagy flux measurements can provide direct evidence of the dynamic process of autophagy. However, few previous studies have provided their evidence based on the measurements of autophagy flux. In contrast with most previous studies, our study showed that increasing age lead to a trend of increased autophagy in skeletal muscle, using autophagy flux measurements. The finding of our study is in consistent with a recent study, which showed that autophagy flux in skeletal muscle increased during aging, whereas chronic contractile activity decreased autophagy flux in the skeletal muscle of the aged mice [12]. The researchers speculated that autophagy increased to selectively remove the dysfunctional organelles so as to maintain a healthy pool of organelles in the skeletal muscle during aging, which resulted in the loss of muscle cell content and muscle mass [12].

Nrf2 is a cyto-protective gene which plays a key role in antioxidant and cell defense. In recent years, increasing studies have revealed a correlation between Nrf2 and autophagy. Pajares et al. showed that Nrf2 activated the expression of autophagy-related genes in mouse brain and embryo fibroblasts [19]. Tang et al. showed that Nrf2 increased the expression of autophagy-related genes in the mice brain in an age dependent manner [20]. Currently, few studies have investigated the association of autophagy with Nrf2 in the muscle tissues [21]. Kitaoka et al. showed that Nrf2 KO did not affect denervation-induced alterations of autophagy in mice skeletal muscle [21]. However, none of these studies have measured autophagy flux in an animal model. Our study showed that Nrf2 KO decreased the levels of autophagy-related proteins, including LC3b-II, LC3b-II/I, P62, Bnip3, and Lamp-1, which was in consistent with previous studies [19, 20]. However, the explanation of our results was different from previous studies. Using autophagy flux measurements, we proved that Nrf2 KO significantly increased autophagy in the muscle tissue. Therefore, we attributed the decreased levels of autophagy-related proteins in the Nrf2 KO mice to the faster clearance of these proteins by autophagy.

In the present study, we measured the expression of autophagy-related proteins both in the static state (Figures 1–5) and in the dynamic state by using autophagy flux measurements (Figures 6–8). Although the difference was not always statistically significant in all the models, these two parts of results supplemented each other to prove the hypothesis. For example, in Figures 3–5, we showed that Nrf2 deficiency caused a trend of decrease in LC3b-II, LC3b-II/LC3b-I, P62, Bnip3, and Lamp-1 in the young, middle-aged, and old mice. Although the difference was not always statistically significant, the trend was consistent in all the three age groups. Moreover, in Figures 7–8, we showed that Nrf2 deficiency increased autophagy flux, both in the young and old mice. Increased autophagy flux can cause a faster clearance of autophagy-related proteins and result in a decreased expression level of these proteins in the static state. Taken together, we concluded that autophagy was increased by Nrf2 KO in mice skeletal muscle.

AMP-activated protein kinase (AMPK) and reactive oxygen species (ROS) are well recognized triggers of autophagy. Our study showed that both AMPK and ROS were upregulated following Nrf2 KO and increasing age, this trend was in consistent with the increasing trend of autophagy flux following Nrf2 KO and increasing age. Therefore, we speculated that Nrf2 KO and increasing age may activate AMPK and ROS signals, which cause excessive activation of autophagy in skeletal muscle. AMPK is activated by an elevated AMP/ATP ratio due to cellular and environmental stress. It has been proved that Nrf2 deficiency impaired mitochondrial membrane potential and respiration and reduced the efficiency of oxidative phosphorylation and the synthesis of ATP [22]. This can partially explain the higher AMPK phosphorylation level in the Nrf2 KO mice. Our study also found that ROS levels were higher in the skeletal muscle of Nrf2 KO mice compared with that in the WT mice, which was in consistent with several previous studies [18, 23]. There is a close relationship between ROS and autophagy. On the one hand, ROS triggers autophagy. On the other hand, cellular ROS level is decreased by the autophagy process, which forms a negative feedback system [24–26]. During the aging process, mitochondrial function and antioxidant systems degenerate in the skeletal muscle cells, and this process is exacerbated by Nrf2 deficiency, which leads to energy metabolism dysfunction and oxidative stress. As a result, AMPK and ROS signals are upregulated to activate the autophagy process. The dysfunctional organelles and proteins were then degraded by autophagy to maintain a healthy pool of organelles and to provide the substrate for energy metabolism, which in turn, limit the overactivation of the AMPK and ROS signals. As a result, muscle cell contents and muscle mass were consumed, which lead to the development of sarcopenia.

Previously, most literature held the opinion that aging results in impaired function of Nrf2 in skeletal muscle [18, 27], which was based on studies in other tissues, such as *Macaca mulatta* vascular endothelial/smooth muscle [28], rat kidney [29], and mice myocardial cells [23]. However, several previous studies have investigated the effect of aging on the expression of Nrf2 and its downstream cytoprotective genes in the skeletal muscle but the results were inconsistent [27, 30]. In recent years, increasing studies have recognized that expression of Nrf2 and its downstream genes in the skeletal muscle can be activated by physical exercise [27, 31]. Safdar et al. found that elderly humans who have a physically active lifestyle have an even higher expression level of Nrf2 and its downstream cytoprotective proteins compared with young subjects [27]. This can partially explain the inconsistent previous studies regarding the effect of aging on the expression of Nrf2 and its downstream cytoprotective genes. Therefore, physical exercise can be an effective therapy for improving the Nrf2 function in the skeletal muscle of elderly.

In conclusion, our study demonstrated that Nrf2 deficiency promoted the increasing trend of autophagy during aging in skeletal muscle. Nrf2 deficiency and increasing age may activate AMPK and ROS signals to cause excessive autophagy in skeletal muscle, which can be a potential mechanism for the development of sarcopenia.

## MATERIALS AND METHODS

### Animal

Nrf2 knockout (KO) mice and their age-matched wild-type (WT) mice at young (5-6 months), middle-aged (11-13 months), and old (20-24 months) age were used in this study. All mice were individually housed under specific pathogen-free facilities with standard environment, diet and water. All of the animal experiments were conducted in accordance with the Guide for the Care and Use of Laboratory Animals, and the study was approved by Institutional Animal Committee of Tongji University.

### Western blot analysis

Gastrocnemius muscle samples were homogenized using tissue lysis buffer (Beyotime Biotechnology, China) containing protease inhibitor cocktail and PhosSTOP phosphatase inhibitors (Roche, Switzerland) in an electric homogenizer. Muscle tissue extracts were obtained by centrifugation at 12000rpm for 20min at 4°C. After quantification of protein concentrations, 20μg of protein samples were separated on sodium dodecyl sulfate-polyacrylamide gel electrophoresis (SDS-PAGE) by electrophoresis, followed by electrotransferring onto a polyvinylidene difluoride (PVDF) membrane (Millipore, USA) in a wet transfer system (Bio-Rad, USA). The membranes were blocked with 5.0% skim milk or 3% bovine serum albumin (BSA) in room temperature for 1 h and subsequently incubated with primary antibodies overnight at 4°C. The membranes were then incubated with horseradish peroxidase (HRP)-conjugated secondary antibodies for 1.5 h at room temperature, followed by image exposure using an enhanced chemiluminescence HRP substrate detection kit (ThermoFisher, USA) under Amersham imager 600 (GE, USA) system. The quantification of proteins was performed by calculating the protein band density of each sample and adjusting to the loading control, using Image J software. The primary antibodies used in this study were anti-LC3b rabbit monoclonal antibody (Abcam, UK), anti-P62 rabbit monoclonal antibody (Abcam, UK), anti-Bnip3 rabbit monoclonal antibody (Abcam, UK), anti-Lamp-1 rabbit polyclonal antibody (Abcam, UK), anti-AMPK rabbit monoclonal antibody (CST, UK), anti-AMPK Thr172 rabbit monoclonal antibody (CST, UK).

### Measurement of autophagy flux in skeletal muscle

Colchicine (COL) was used as autophagy inhibitor. Young WT, young KO, old WT and old KO mice were randomly assigned to treatment or control groups. Mice in the treatment group received intraperitoneal injections of sterile solutions of colchicine (0.4 mg/kg/day; Sigma) in 3 consecutive days, whereas mice in the control group received injection of equal doses of saline. Protein levels of LC3b, P62, Bnip3, and Lamp-1 were measured in gastrocnemius extracts by western-blot. The ratio of COL values/mean CON values were used to reflect the autophagosome flux values (e.g. young WT COL/mean young WT CON) in each groups.

### In situ reactive oxygen species (ROS) detection

Tibialis anterior muscle samples were embedded in the OCT solution (TissueTek, Japan) and frozen in -80 °C. Frozen sections were made using a freezing microtome and incubated with 5μM dihydroethidium (DHE) and 200nM Mito-Tracker green in 37°C for 30min followed 3 washes with PBS for 5min each time. Immunofluorescence was viewed with a fluorescence microscope (Leica, Germany). Fluorescence intensity of the images was analyzed using Image J software.

### Hematoxylin and eosin (HE) staining of skeletal muscle and measurement of myofibril cross-sectional area (CSA)

Extensor digitorum longus (EDL) and soleus (SOL) muscle samples were fixed in 4% paraformaldehyde, embedded in paraffin, sectioned and stained with hematoxylin and eosin. The sections were observed and the images were captured under a light microscope system (Leica, Germany). Myofibril cross-sectional area (CSA) was calculated by quantification of the mean CSA of 100 fibers in each mouse using Image J software.

### Statistical analysis

Continuous variables were represented as mean and stand error (SE). Expression levels of proteins in western-blot analysis were compared using Student’s t-tests or one-way ANOVA. Reactive oxygen species (ROS) levels and CSA were compared using two-way ANOVA followed by Bonferroni post-hoc analysis. Two-tailed P values <0.05 were considered significant. GraphPad Prism software (GraphPad Software, USA) and SPSS statistics version 22.0 (IBM, USA) were used for the statistical analysis.

## References

[1] Masiero E, Agatea L, Mammucari C, Blaauw B, Loro E, Komatsu M, Metzger D, Reggiani C, Schiaffino S, Sandri M. Autophagy is required to maintain muscle mass. Cell Metab. 2009; 10:507–15. 10.1016/j.cmet.2009.10.008 19945408

[2] Chen H, Vermulst M, Wang YE, Chomyn A, Prolla TA, McCaffery JM, Chan DC. Mitochondrial fusion is required for mtDNA stability in skeletal muscle and tolerance of mtDNA mutations. Cell. 2010; 141:280–89. 10.1016/j.cell.2010.02.026 20403324 PMC2876819

[3] Petrovski G, Das DK. Does autophagy take a front seat in lifespan extension? J Cell Mol Med. 2010; 14:2543–51. 10.1111/j.1582-4934.2010.01196.x 21114762 PMC4373474

[4] Fan J, Kou X, Jia S, Yang X, Yang Y, Chen N. Autophagy as a Potential Target for Sarcopenia. J Cell Physiol. 2016; 231:1450–59. 10.1002/jcp.25260 26580995

[5] Cruz-Jentoft AJ, Baeyens JP, Bauer JM, Boirie Y, Cederholm T, Landi F, Martin FC, Michel JP, Rolland Y, Schneider SM, Topinková E, Vandewoude M, Zamboni M, and European Working Group on Sarcopenia in Older People. Sarcopenia: European consensus on definition and diagnosis: Report of the European Working Group on Sarcopenia in Older People. Age Ageing. 2010; 39:412–23. 10.1093/ageing/afq034 20392703 PMC2886201

[6] Escobar KA, Cole NH, Mermier CM, VanDusseldorp TA. Autophagy and aging: maintaining the proteome through exercise and caloric restriction. Aging Cell. 2019; 18:e12876. 10.1111/acel.12876 30430746 PMC6351830

[7] López-Otín C, Blasco MA, Partridge L, Serrano M, Kroemer G. The hallmarks of aging. Cell. 2013; 153:1194–217. 10.1016/j.cell.2013.05.039 23746838 PMC3836174

[8] Alvers AL, Fishwick LK, Wood MS, Hu D, Chung HS, Dunn WA Jr, Aris JP. Autophagy and amino acid homeostasis are required for chronological longevity in Saccharomyces cerevisiae. Aging Cell. 2009; 8:353–69. 10.1111/j.1474-9726.2009.00469.x 19302372 PMC2802268

[9] Hars ES, Qi H, Ryazanov AG, Jin S, Cai L, Hu C, Liu LF. Autophagy regulates ageing in C. elegans. Autophagy. 2007; 3:93–95. 10.4161/auto.3636 17204841

[10] Jiao J, Demontis F. Skeletal muscle autophagy and its role in sarcopenia and organismal aging. Curr Opin Pharmacol. 2017; 34:1–6. 10.1016/j.coph.2017.03.009 28407519

[11] Sebastián D, Sorianello E, Segalés J, Irazoki A, Ruiz-Bonilla V, Sala D, Planet E, Berenguer-Llergo A, Muñoz JP, Sánchez-Feutrie M, Plana N, Hernández-Álvarez MI, Serrano AL, et al. Mfn2 deficiency links age-related sarcopenia and impaired autophagy to activation of an adaptive mitophagy pathway. EMBO J. 2016; 35:1677–93. 10.15252/embj.201593084 27334614 PMC4969577

[12] Carter HN, Kim Y, Erlich AT, Zarrin-Khat D, Hood DA. Autophagy and mitophagy flux in young and aged skeletal muscle following chronic contractile activity. J Physiol. 2018; 596:3567–84. 10.1113/JP275998 29781176 PMC6092298

[13] McMullen CA, Ferry AL, Gamboa JL, Andrade FH, Dupont-Versteegden EE. Age-related changes of cell death pathways in rat extraocular muscle. Exp Gerontol. 2009; 44:420–25. 10.1016/j.exger.2009.03.006 19341788 PMC2720059

[14] Komatsu M, Waguri S, Ueno T, Iwata J, Murata S, Tanida I, Ezaki J, Mizushima N, Ohsumi Y, Uchiyama Y, Kominami E, Tanaka K, Chiba T. Impairment of starvation-induced and constitutive autophagy in Atg7-deficient mice. J Cell Biol. 2005; 169:425–34. 10.1083/jcb.200412022 15866887 PMC2171928

[15] Jiang T, Harder B, Rojo de la Vega M, Wong PK, Chapman E, Zhang DD. p62 links autophagy and Nrf2 signaling. Free Radic Biol Med. 2015; 88:199–204. 10.1016/j.freeradbiomed.2015.06.014 26117325 PMC4628872

[16] Zuo R, Wang Y, Li J, Wu J, Wang W, Li B, Sun C, Wang Z, Shi C, Zhou Y, Liu M, Zhang C. Rapamycin Induced Autophagy Inhibits Inflammation-Mediated Endplate Degeneration by Enhancing Nrf2/Keap1 Signaling of Cartilage Endplate Stem Cells. Stem Cells. 2019; 37:828–40. 10.1002/stem.2999 30840341

[17] Huang DD, Fan SD, Chen XY, Yan XL, Zhang XZ, Ma BW, Yu DY, Xiao WY, Zhuang CL, Yu Z. Nrf2 deficiency exacerbates frailty and sarcopenia by impairing skeletal muscle mitochondrial biogenesis and dynamics in an age-dependent manner. Exp Gerontol. 2019; 119:61–73. 10.1016/j.exger.2019.01.022 30690066

[18] Miller CJ, Gounder SS, Kannan S, Goutam K, Muthusamy VR, Firpo MA, Symons JD, Paine R 3rd, Hoidal JR, Rajasekaran NS. Disruption of Nrf2/ARE signaling impairs antioxidant mechanisms and promotes cell degradation pathways in aged skeletal muscle. Biochim Biophys Acta. 2012; 1822:1038–50. 10.1016/j.bbadis.2012.02.007 22366763

[19] Pajares M, Jiménez-Moreno N, García-Yagüe AJ, Escoll M, de Ceballos ML, Van Leuven F, Rábano A, Yamamoto M, Rojo AI, Cuadrado A. Transcription factor NFE2L2/NRF2 is a regulator of macroautophagy genes. Autophagy. 2016; 12:1902–16. 10.1080/15548627.2016.1208889 27427974 PMC5079676

[20] Tang M, Ji C, Pallo S, Rahman I, Johnson GV. Nrf2 mediates the expression of BAG3 and autophagy cargo adaptor proteins and tau clearance in an age-dependent manner. Neurobiol Aging. 2018; 63:128–39. 10.1016/j.neurobiolaging.2017.12.001 29304346 PMC5801049

[21] Kitaoka Y, Takeda K, Tamura Y, Fujimaki S, Takemasa T, Hatta H. Nrf2 deficiency does not affect denervation-induced alterations in mitochondrial fission and fusion proteins in skeletal muscle. Physiol Rep. 2016; 4:4. 10.14814/phy2.13064 28039408 PMC5210374

[22] Dinkova-Kostova AT, Abramov AY. The emerging role of Nrf2 in mitochondrial function. Free Radic Biol Med. 2015; 88:179–88. 10.1016/j.freeradbiomed.2015.04.036 25975984 PMC4726722

[23] Gounder SS, Kannan S, Devadoss D, Miller CJ, Whitehead KJ, Odelberg SJ, Firpo MA, Paine R 3rd, Hoidal JR, Abel ED, Rajasekaran NS. Impaired transcriptional activity of Nrf2 in age-related myocardial oxidative stress is reversible by moderate exercise training. PLoS One. 2012; 7:e45697. 10.1371/journal.pone.0045697 23029187 PMC3454427

[24] Li L, Tan J, Miao Y, Lei P, Zhang Q. ROS and Autophagy: Interactions and Molecular Regulatory Mechanisms. Cell Mol Neurobiol. 2015; 35:615–21. 10.1007/s10571-015-0166-x 25722131 PMC11486209

[25] Van Erp AC, Hoeksma D, Rebolledo RA, Ottens PJ, Jochmans I, Monbaliu D, Pirenne J, Leuvenink HGD, Decuypere JP. The Crosstalk between ROS and Autophagy in the Field of Transplantation Medicine. Oxid Med Cell Longev. 2017; 2017:7120962. 10.1155/2017/7120962 29410735 PMC5749284

[26] Scherz-Shouval R, Elazar Z. Regulation of autophagy by ROS: physiology and pathology. Trends Biochem Sci. 2011; 36:30–38. 10.1016/j.tibs.2010.07.007 20728362

[27] Safdar A, deBeer J, Tarnopolsky MA. Dysfunctional Nrf2-Keap1 redox signaling in skeletal muscle of the sedentary old. Free Radic Biol Med. 2010; 49:1487–93. 10.1016/j.freeradbiomed.2010.08.010 20708680

[28] Ungvari Z, Bailey-Downs L, Gautam T, Sosnowska D, Wang M, Monticone RE, Telljohann R, Pinto JT, de Cabo R, Sonntag WE, Lakatta EG, Csiszar A. Age-associated vascular oxidative stress, Nrf2 dysfunction, and NF-{kappa}B activation in the nonhuman primate Macaca mulatta. J Gerontol A Biol Sci Med Sci. 2011; 66:866–75. 10.1093/gerona/glr092 21622983 PMC3148762

[29] Asghar M, George L, Lokhandwala MF. Exercise decreases oxidative stress and inflammation and restores renal dopamine D1 receptor function in old rats. Am J Physiol Renal Physiol. 2007; 293:F914–19. 10.1152/ajprenal.00272.2007 17634393

[30] Kitaoka Y, Tamura Y, Takahashi K, Takeda K, Takemasa T, Hatta H. Effects of Nrf2 deficiency on mitochondrial oxidative stress in aged skeletal muscle. Physiol Rep. 2019; 7:e13998. 10.14814/phy2.13998 30756520 PMC6372533

[31] Islam H, Bonafiglia JT, Turnbull PC, Simpson CA, Perry CG, Gurd BJ. The impact of acute and chronic exercise on Nrf2 expression in relation to markers of mitochondrial biogenesis in human skeletal muscle. Eur J Appl Physiol. 2020; 120:149–60. 10.1007/s00421-019-04259-7 31707475

